# When Top-Down Becomes Bottom Up: Behaviour of Hyperdense Howler Monkeys (*Alouatta seniculus*) Trapped on a 0.6 Ha Island

**DOI:** 10.1371/journal.pone.0082197

**Published:** 2014-04-17

**Authors:** Gabriela Orihuela, John Terborgh, Natalia Ceballos, Kenneth Glander

**Affiliations:** 1 Fairchild Tropical Botanical Garden, Coral Gables, Florida, United States of America; 2 Center for Tropical Conservation, Nicholas School of the Environment and Earth Sciences, Duke University, Durham, North Carolina, United States of America; 3 Wildlife Research Group, The Anatomy School, University of Cambridge, Cambridge, United Kingdom; 4 Department of Evolutionary Anthropology, Duke University, Durham, North Carolina, United States of America; Universidade de São Paulo, Faculdade de Filosofia Ciências e Letras de Ribeirão Preto, Brazil

## Abstract

Predators are a ubiquitous presence in most natural environments. Opportunities to contrast the behaviour of a species in the presence and absence of predators are thus rare. Here we report on the behaviour of howler monkey groups living under radically different conditions on two land-bridge islands in Lago Guri, Venezuela. One group of 6 adults inhabited a 190-ha island (Danto) where they were exposed to multiple potential predators. This group, the control, occupied a home range of 23 ha and contested access to food resources with neighbouring groups in typical fashion. The second group, containing 6 adults, was isolated on a remote, predator-free 0.6 ha islet (Iguana) offering limited food resources. Howlers living on the large island moved, fed and rested in a coherent group, frequently engaged in affiliative activities, rarely displayed agonistic behaviour and maintained intergroup spacing through howling. In contrast, the howlers on Iguana showed repulsion, as individuals spent most of their time spaced widely around the perimeter of the island. Iguana howlers rarely engaged in affiliative behaviour, often chased or fought with one another and were not observed to howl. These behaviors are interpreted as adjustments to the unrelenting deprivation associated with bottom-up limitation in a predator-free environment.

## Introduction

Top-down and bottom-up regulated populations lie at the poles of a spectrum of possibilities. Under the top-down scenario, predators regulate prey numbers such that prey populations are maintained at densities below carrying capacity, and food does not limit numbers. Under bottom-up regulation, predation is absent and the population is free to increase until it reaches carrying capacity, at which point it becomes limited by its food supply [1]. Since virtually all ecosystems contain predators, and predator removal is experimentally difficult, the bottom-up condition is little investigated [2]. In particular, there are relatively few species for which the normal density and behaviour of individuals can be contrasted with density and behaviour at carrying capacity [3]. Are ‘normal’ densities near or far from carrying capacity? In general, the answer is not known.

The special circumstances created by the formation of a 4,300 km^2^ hydroelectric impoundment, Lago Guri, in the State of Bolivar, Venezuela [4], enabled us to investigate the ecology and behaviour of a social herbivore, the red howler monkey (*Alouatta seniculus*, hereafter simply howler), in two contrasting situations: 1) a ‘normal’ population on a large 190 ha island (Danto), and 2) a predator-free population living at ultrahigh density on a tiny 0.6 ha island (Iguana). The two howler populations became isolated in 1986 when water rising behind the Raul Leoní dam created hundreds of land-bridge islands ranging in size from <<1 ha to ca 750 ha [5].

Although group sizes were similar on both the small and large islands, the circumstances of the groups were markedly different. Danto howlers occupied a normal-sized home range containing thousands of trees (minimum polygon of 23 ha), whereas the 0.6 ha available to Iguana howlers supported only 351 trees ≥10 cm dbh and many fewer tree species [5]. In the limited area offered by Iguana, howler density was equivalent to 1000/km^2^, or more than 10 times that of howlers on Danto or howlers living in similar dry forest habitat on the Venezuelan mainland [6], [7], [8], [9]. In their larger home range, Danto howlers harvested a wide range of resources, often from uncommon tree species, that were mostly absent from Iguana [5]. Danto howlers frequently ingested fruit (22% of feeding time), a preferred resource [10] that was almost entirely lacking (<2%) in the diets of Iguana howlers. Iguana howlers were thus compelled to subsist on a diet of less-preferred resources that, although available to the howlers on Danto, were used little or not at all by them [5]. The behaviour of howlers in these two situations was dramatically different, as shall be detailed below.

## Methods

We observed groups of howlers on both islands in 1999 and 2000. Located <1 km from the mainland, Danto supported or was occasionally visited by several predators at least potentially capable of preying on howlers: jaguar (*Panthera onca*), puma (*Puma concolor*), ocelot (*Leopardis pardalis*), harpy eagle (*Harpia harpyja*), solitary eagle (*Harpyhaliaetus solitarius*), boa constrictor (*Constrictor constrictor*), anaconda (*Eunectes murinus*) [2]. In contrast, Iguana was situated in the middle of the impoundment, 7 km from the mainland, and appeared to offer a predator-free environment, for in 12 years of using Iguana as a base camp, our group never observed a predator capable of taking a large mammal [2].

The Danto group contained six adults and subadults both years, accompanied by 2 or 3 infants and/or juveniles. The Iguana group contained 6 individuals in both years (five adults and subadults and one juvenile). Both islands were covered in semi-evergreen tropical dry forest dominated by legumes and containing ca 50–70 species of trees per ha [5]. Censused annually over a period of 9 years (1993–2002), the Iguana group was observed to be self-maintaining, experiencing both births and deaths, yet persisting at almost constant numbers [6].

Howlers confined to tiny Lago Guri islets proved to be extremely shy and several years of persistent effort were required to habituate the Iguana group. Once habituation was adequate, we conducted observations using dawn-to-dark follows and continuous sampling of feeding and behaviour, following [11]. All observations were conducted between late May and early July in the local rainy season. Contact at Danto summed to 74 and 146 hours (total 220) in 1999 and 2000, respectively, and to 106 and 101 hours (total 207) at Iguana. The behaviors reported below were recorded *ad libitum*.

To assess the condition of howlers living at abnormally high densities, we captured and weighed 8 adult individuals (4 males, 4 females) taken from 5 small (<1 ha) Lago Guri islands in August, 2001. Monkeys were captured using the method described in [12]. Briefly, they were darted with Telazol and fell into a sheet being held taught by two assistants so that they landed softly. The islands from which howlers were removed did not include Iguana.

Statistical evaluations of categorical behaviours were conducted via Mann-Whitney U test and the analysis of weight differences between howlers captured on small Lago Guri islands and the Venezuelan mainland were conducted using ANOVA.

### Ethics statement

Captured monkeys were examined and released into the wild within a few hours of capture. They were not caged or fed and proved virtually free of parasites, so no treatment was administered. After inspection and weighing, they were transported across 5–7 km of open water while recovering from anesthesia and released on the nearby mainland into the forested watershed protection zone of the Guri impoundment. They were handled gently at all times. Duke University Institutional Animal Care and Use Committee approved the capture protocol, including capture, handling, measuring and relocation, under #AO73-96-2. EDELCA, the hydroelectric company that managed the Guri impoundment granted permission to capture and release the animals.

## Results

Howlers typically live in cohesive groups led by a dominant adult male. The members of such groups travel, feed and rest together and display little agonistic behaviour *inter se*, though dominant males are aggressive toward their counterparts in neighbouring groups [8], [13]. The howlers of Danto behaved in typical fashion. Group members travelled, fed and rested in close proximity and displayed little agonistic behaviour *inter se*. Danto howlers shared overlapping home ranges with neighbouring groups (degree of overlap not quantified) and frequently contested possession of resource-bearing trees with loud howling matches. Agonistic behaviour was thus almost entirely restricted to between-group, rather than within-group, interactions.

Confined to a home range less than 5% of the typical area for the species, and isolated from other conspecifics, Iguana howlers exhibited radically different behaviour (Table 1). Instead of living in a coherent group, they distributed themselves centrifugally around the island. Individuals, except a female with a dependent juvenile, spent much of their time alone. Encounters took place most often in resource trees, where agonistic interactions were the norm. The dominant male habitually occupied and defended what we took to be the most productive tree available. Other individuals were allowed to feed in this tree only after the dominant male had moved away and then usually according to a hierarchy in which adult females took precedence over juveniles. Subordinate individuals often did not attempt to feed in trees used by more dominant individuals.

**Table 1:** Behaviors of howler monkeys on large (Danto - 190 ha) and small (Iguana - 0.6 ha) islands in Lago Guri, Venezuela in 1999 and 2000 (number of occurrences of the behavior per 100 contact hours).

	No. contact hours	Allogrooming	Playing	Fighting	Chasing	Pushing away	Howling bouts
Danto Machado 1999	72	21	6	1	0	0	0
Danto Machado 2000	146	37	22	0	4	2	8
Per 100 contact hrs	218	26.6	12.8	0.5	1.8	0.9	5.5
Iguana 1999	106	0	0	3	3	1	0
Iguana 2000	101	1	0	4	2	2	0
Per 100 contact hrs	207	0.5	0.0	3.4	2.4	1.4	0.0
P-value (two-tailed)^1^		<0.0001	<0.0001	0.013	0.492	0.751	0.006
1. Mann-Whitney U							

In the absence of encounters with other groups, Iguana howlers did not howl, although the dominant male showed clear interest in the howling of other males on neighbouring islands by raising his head at the onset of a howling bout and orienting toward the source of the sound.

Frequent conflicts over access to resource trees generated high levels of within-group conflict among Iguana howlers (Table 1). Most agonistic encounters took the form of chases in which one individual expelled another from a resource tree. Chases were also observed in the Danto group but they rarely involved physical contact (classified as fighting), whereas fighting was 7 times more frequent among Iguana howlers. Intense avoidance led individuals to seek refuge in widely scattered trees, a practice that largely precluded affiliative behaviours. Thus, allogrooming bouts involving Iguana howlers were only 2% as frequent as in the Danto group. Juveniles also exhibited reduced affiliative behaviour and were not observed to engage in play on Iguana.

Evidence of dietary stress was obtained through the capture of 8 howlers (4 males, 4 females) on 5 small islands (Table 2). After weighing and inspection for parasites, these animals were released on the mainland. The mean weight of the 4 males was 5.4 kg, whereas the mean weight of 4 females was 3.2 kg.

**Table 2:** Mean weights (SD) of adult male and female howlers captured on small Lago Guri islets compared to adults from similar dry forest habitat in the Venezuelan states of Apure [9] and Guárico [18], [19].

	Guri	Apure	Guarico	F, P	F, P	F, P
	A	B	C	A & B	A & C	B & C
Males	N = 4	N = 27	N = 10			
Weight (g)	5364	5973	6500	4.18	6.39	NS
(SD)	(1194)	(440)	(600)	0.008	0.008	
Females	N = 4	N = 22	N = 4			
Weight (g)	3209	4183	4500	22.14	66.32	NS
(SD)	(278)	(402)	(200)	0	0.0007	

F and P refer to the respective F and P values (on successive lines) of the ANOVAS used to conduct the analysis. A & B, A & C, etc. refer to comparisons between columns A, B, and C.

## Discussion

Howlers were trapped on the 0.6 ha island of Iguana in 1986 with the creation of Lago Guri and persisted at a density equivalent to 1000 per km^2^ through 2002 when we last observed them. Such high densities are unknown on the nearby Venezuelan mainland where the same species normally occurs at densities of <100 per km^2^ [6], [7], [8]. An evident lack of predation facilitated high survival and persistence at a density >10 times greater than normal, suggesting that Iguana howlers were limited from the bottom-up by food availability. In contrast, large-island (Danto) howlers lived at a much lower conspecific density in a home range of ≥23 ha [5]. Whether the lower density of howlers on Danto was due to predation, infanticide, competition from other primate species or some other factor could not be determined. Regardless, with access to larger home ranges, Danto howlers utilized a wider range of resources than those of Iguana, including many resource species not present on Iguana, consumed more fruit (22% of feeding time vs. 2%) and less foliage (55% vs. 73%) [5].

Persistence of Iguana howlers for 16 years (1986–2002) at a density equivalent to 1000 individuals/km^2^ suggests that the carrying capacity of the dry forest habitat is much greater than that suggested by the densities considered normal for the species in this habitat [7], [8], [9]. Persistence of howlers at such a high density for so long is all the more remarkable in light of the fact that the howlers of Iguana shared the island with hyperdense populations of two additional generalist folivores, the common iguana (*Iguana iguana*) and leaf-cutter ants (*Atta* spp.) [14]. Alternatively, the Iguana howler population may not have been at carrying capacity during the period of our observations but rather in demographic overshoot, as repeated defoliation of resource trees was associated with high tree mortality (2). In either case, the much lower densities typical of mainland howler populations and the avoidance by Danto howlers of important resources utilized by Iguana howlers suggest that howler populations subject to predators, infanticide and perhaps other mortality factors are not ordinarily food limited. However, the true value of carrying capacity in density units remains elusive. Nevertheless, the observations reported here suggest that carrying capacity is probably a density several to many times higher than the densities normally observed in mainland populations.

Crowding and food deprivation among Iguana howlers was associated with extraordinary differences in behavior in comparison to the Danto group that lived under more normal circumstances. Resource scarcity plausibly led to increased within-group competition for food resources among Iguana howlers [5], [15]. Elevated levels of chasing and fighting and the centrifugal dispersion of individuals may also have been responses to resource scarcity. Individuals that engaged in frequent contests for food resources tended to avoid higher-ranking group members and to scatter around the periphery of the island. Such anti-social behaviour is contrary to the close grouping one typically sees in social vertebrates under the threat of predation [16]. Lack of play in Iguana juveniles may have reflected reduced opportunity as well as lower activity levels associated with dietary stress, as Iguana howlers rested more and travelled less than their counterparts on Danto.

Hierarchical access to food resources may lie behind the observation that the body weights of males from tiny islands similar to Danto were closer to those of males living on the mainland (88%) than was the case with females (76%). Iguana females also manifested a very low reproductive rate of 0.125 birth per female-year vs. 0.5 per female-year for Danto females [6].

Confinement to a tiny isolated island carries with it the absence of any threat of group takeovers by unrelated males and associated infanticide [17], [18] and plausibly explains the absence of howling.

Howlers occupying small islands in Lago Guri live in a world regulated from the bottom up. Underweight, perpetually intimidated by higher-ranking individuals and obliged to subsist on non-preferred food resources, they live lives of social and caloric deprivation [3], [5]. Thus, there is little to be envied about a bottom-up world other than freedom from predation. Under normal circumstances, howlers experience a constant threat of predation but enjoy the benefits of a relative abundance of food resources, which, in turn, allows amity and social coherence. Might there be a lesson for us humans here?
